# Supplementary material for: Soil Microbial Co-Occurrence Patterns under Controlled-Release Urea and Fulvic Acid Applications
Source: Microorganisms. 2022 Sep 12;10(9):1823. doi: 10.3390/microorganisms10091823 (PMC9502011; doi:10.3390/microorganisms10091823)
Supplement: Supplementary file 1 [file microorganisms-10-01823-s001.zip › microorganisms-1865375-supplementary.pdf]

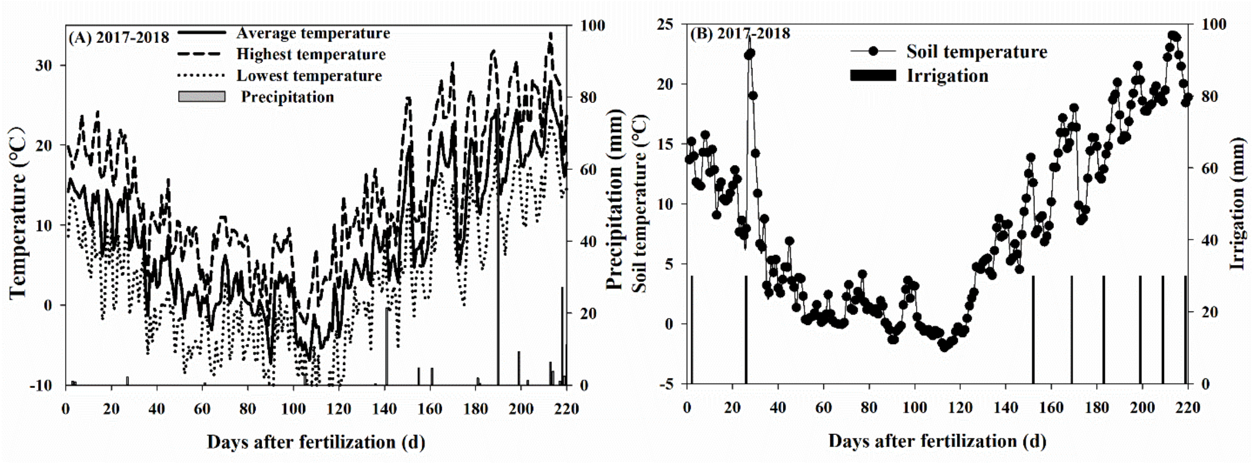

Supplementary Figure S1. Meteorological data (air temperature, precipitation and irrigation) in this field after fertilization during the growing seasons for wheat (October–June 2017-2018) at the experimental site: (A) Air temperature and precipitation, (B) Soil temperature and irrigation.

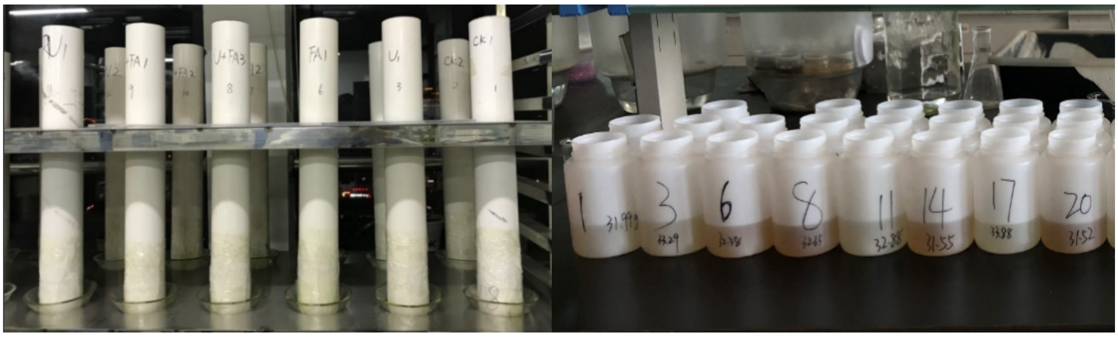

Supplementary Figure S2. The experimental device of soil Column Leaching Experiment.

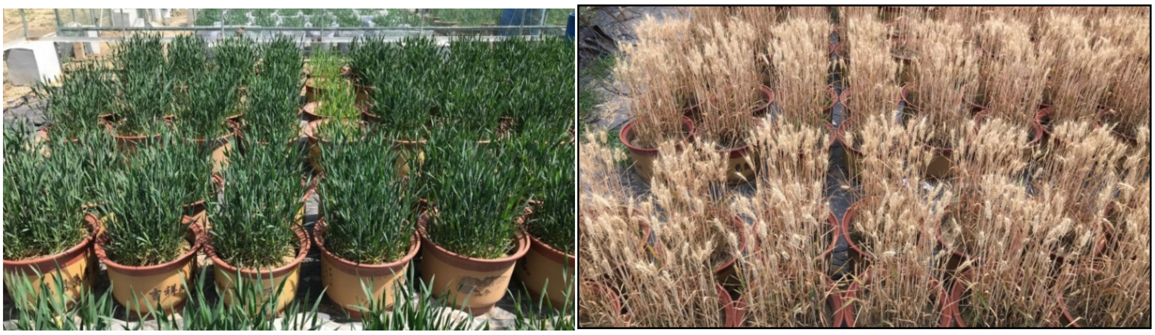

Supplementary Figure S3. The experimental process of pot experiment.

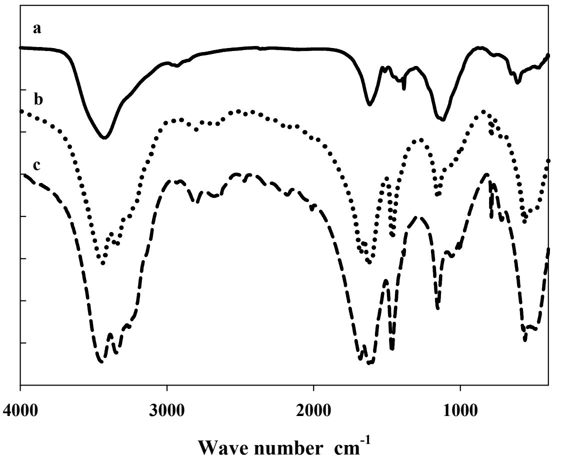

Supplementary Figure S4. Infrared spectrogram of (a) fulvic acid, (b) urea, and (c) urea and fulvic acid.

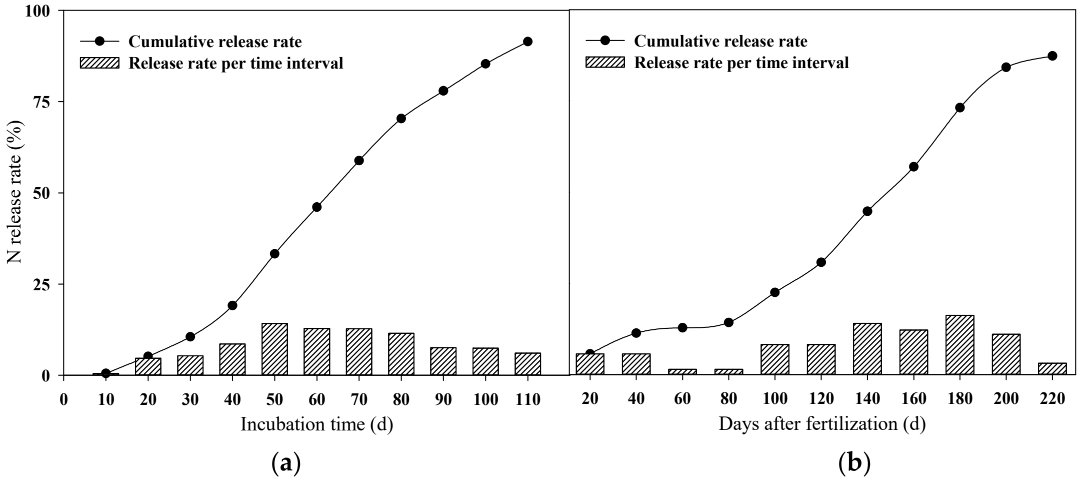

Supplementary Figure S5. Nitrogen release of the controlled release urea, in water at 25°C (a) and in soil under field condition (b).

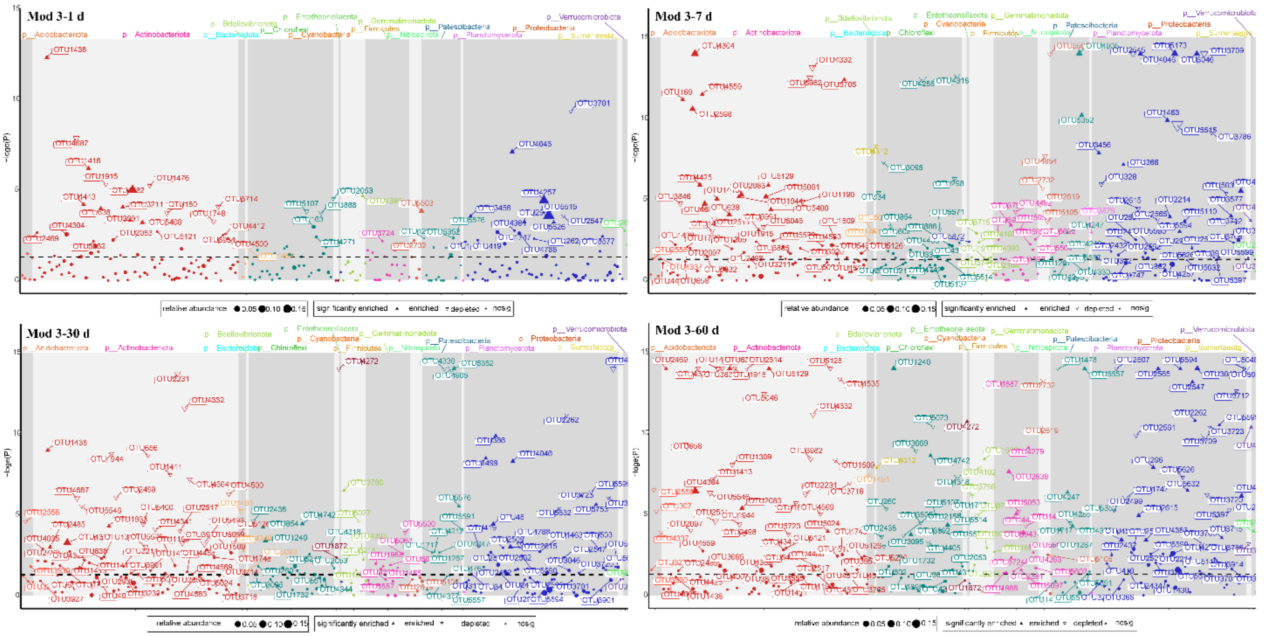

Supplementary Figure S6. Manhattan plots showing OTUs with significant differences in relative

abundance in CRU and CRU+FA treatments in Mod 3 during different fertilizer days. Each triangle or circle represents an individual OTU. Upward solid triangles or downward hollow triangles represent OTUs enriched or depleted, respectively, whereas circles represent OTUs that are not significantly enriched or depleted. The dashed line corresponds to the false discovery rate-corrected threshold P-value for significance ($\alpha = 0.05$). The color of each dot represents the taxonomic affiliation of the OTU (phylum level), and the size corresponds to its relative abundance in the samples.

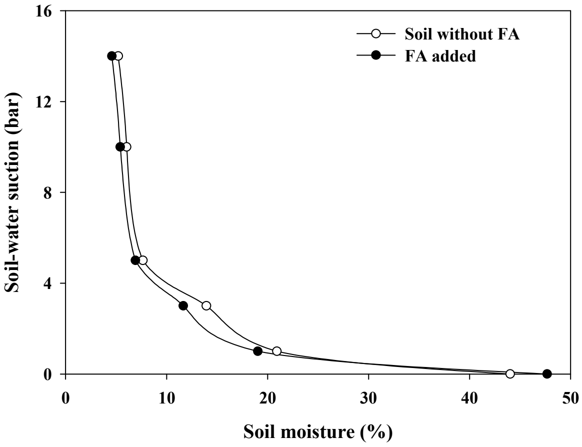

Supplementary Figure S7. Soil-water characteristic curve after adding FA compared to the soil without FA. Treatment components: Control (soil without FA); FA (Fulvic acid 90 kg ha^{-1}).

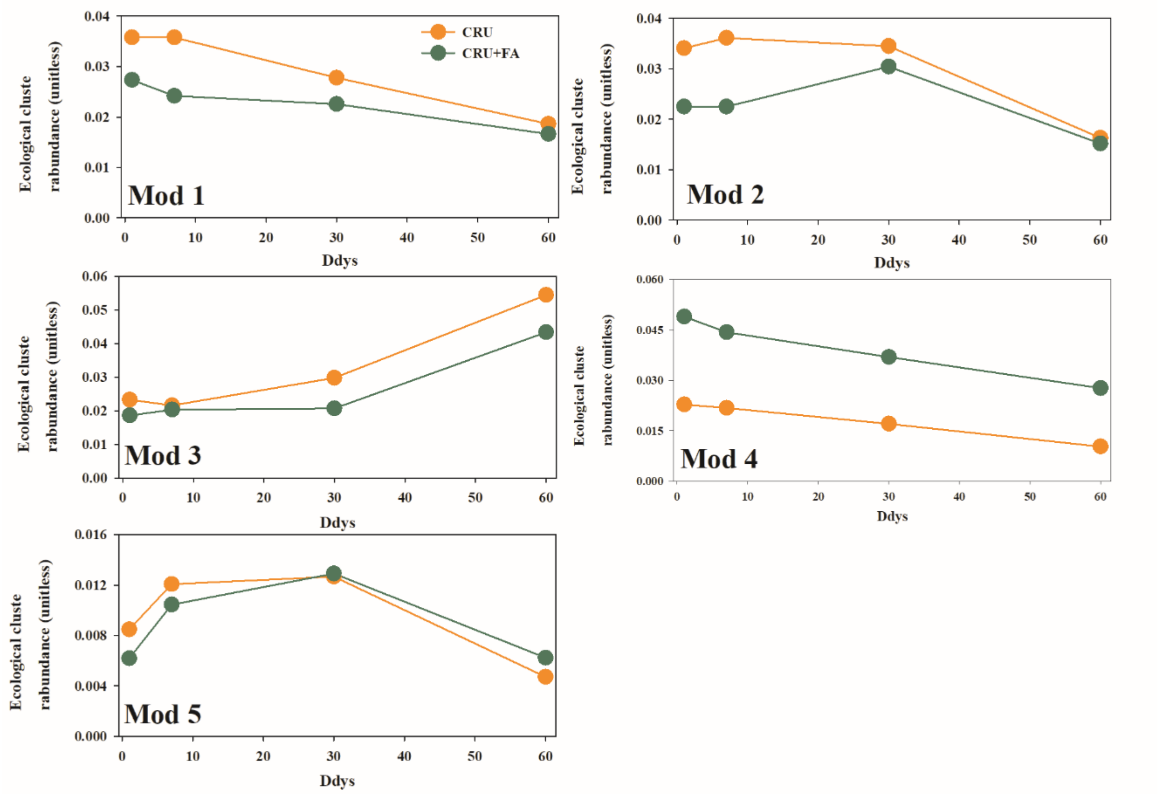

Supplementary Figure S8. The relationship between different days, different treatments and the relative abundance of the selected ecological cluster (Mod 1-5).

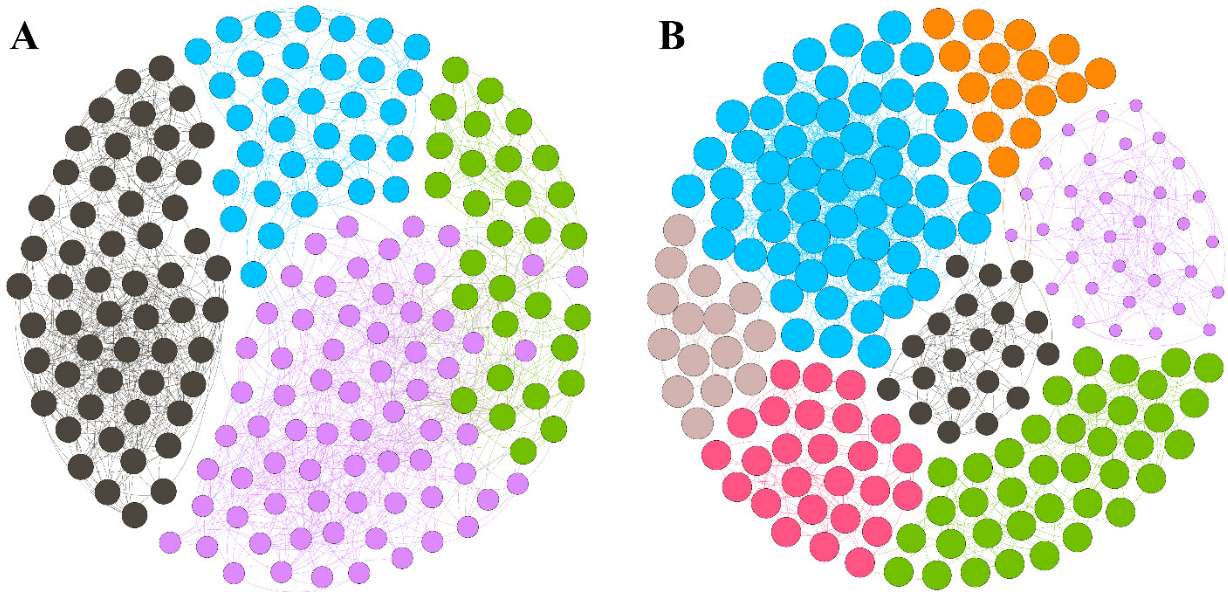

Supplementary Figure S9. Microbial network diagram with nodes (taxa) colored by each of the major ecological clusters (modules, Mod) of bacterial communities from CRU treatment (A), and that from CRU+FA treatment (B).

Supplementary Table S1. The soil nutrients change of the soil microbial incubation, the soil column leaching and the pot experiments.

Treatment[a]	NH_4^+_S	NO_3^-_S	pH_S	NH_4^+_L	NO_3^-_L	HCO_3^-_L	pH_L	NH_4^+_P	NO_3^-_P	pH_P
	mg kg^{-1}			mg mL^{-1}				mg kg^{-1}		
CRU1d	14.9 d[b]	13.8 g	7.0 ab	0.1 c	1.9 b	0.3 b	7. 3 b	7.6 c	15.6 e	7.5 b
CRU7d	11. 7 de	30.2 d	7.3 a	1.3 b	2.2 b	0.1 b	8.1 a	2.5 e	10.3 f	7.6 b
CRU30d	24.1 c	116.8 b	6.4 b	1.7 ab	13.4 a	0.1 b	7.1 b	8.0 c	47.4 c	8.1 a
CRU60d	5.0 f	51.0 c	5.7 bc	1.8 ab	13.7 a	0.1 b	8.1 a	22.3 a	39.6 cd	8.0 a
CRU_FA1d	23.2 c	20.9 f	7.2 ab	0.5 c	0 d	1.7 a	7.8 b	6.9 d	13.2 e	7.5 b
CRU_FA7d	16.1 d	26.5 e	7.6 a	2.1 a	0.4 c	0.1 b	8.3 a	2.0 e	10.1 f	7.6 b
CRU_FA30d	36.0 b	123.1 a	6.1 b	2.1 a	11.9 a	0.1 b	7.8 b	10.8 b	56.7 b	8.0 a
CRU_FA60d	111.9 a	48.7 cd	5.5 c	2.2 a	13.2 a	0.1 b	8.3 a	23.6 a	72.8 a	8.2 a

Note: NO_3^-_L: the nitrate nitrogen from leaching solution; NH_4^+_L: the ammonium nitrogen from leaching solution; HO_3^-_L: the bicarbonate from leaching solution; pH_L: the pH value from leaching solution; NO_3^-_S: the nitrate nitrogen from incubator cultivated experiment; NH_4^+_S: the ammonium nitrogen from incubator cultivated experiment; pH_S: the pH value from incubator cultivated experiment; NO_3^-_P: the nitrate nitrogen from pot experiment; NH_4^+_P: the ammonium nitrogen from pot experiment; pH_P: the pH value from pot experiment.

[a] Treatments: Controlled-release urea (CRU); Controlled-release urea combined with FA (CRU+FA). [b] Means within each column followed by the same letters were not significantly different based on a one-way ANOVA followed by Duncan 's multiple-range test ($P > 0.05$)

Supplementary Table S2. The effects of fertilization treatments on bacterial *beta* diversity.

	Soil bacterial
Different treatments	$F=4.94, R=0.29^{***}$
Different incubation days	$F=9.98, R=0.96^{***}$

Note: Values in table represent the pseudo-F ratio (F), the estimation of the variance component (R) and the level of significance ($^*P < 0.05$, $^{**}P < 0.01$, $^{***}P < 0.001$) for PERMANOVA.
